# Introducing global health into the undergraduate medical school curriculum using an e-learning program: a mixed method pilot study

**DOI:** 10.1186/s12909-015-0421-3

**Published:** 2015-09-02

**Authors:** Douglas Gruner, Kevin Pottie, Douglas Archibald, Jill Allison, Vicki Sabourin, Imane Belcaid, Anne McCarthy, Mahli Brindamour, Lana Augustincic Polec, Pauline Duke

**Affiliations:** 1 Department of Family Medicine, University of Ottawa, 43 Bruyère Street, K1N 5C8 Ottawa, ON Canada; 2 Department of Epidemiology and Community Medicine, University of Ottawa, 451 Smyth Road, K1H 8M5 Ottawa, ON Canada; 3 C.T. Lamont Centre for Research in Primary Care, University of Ottawa, 43 Bruyère Street, K1N 5C8 Ottawa, ON Canada; 4 Division of Community Health and Humanities, Memorial University of Newfoundland, 300 Prince Phillip Drive, A1B 3V6 St. John’s, NF Canada; 5 Department of Medicine, Faculty of Medicine, University of Ottawa, Ottawa Hospital General Campus, 501 Smyth Road, K1H 8L6 Ottawa, ON Canada; 6 Office of Global Health, Faculty of Medicine, University of Ottawa, 451 Smyth Road, K1H 8M5 Ottawa, ON Canada; 7 Department of Paediatrics, University of Saskatchewan, Royal University Hospital, 103 Hospital Drive, S7N 0W8 Saskatoon, SK Canada; 8 Department of Family Medicine, Memorial University of Newfoundland, 300 Prince Phillip Drive, A1B 3V6 St. John’s, NF Canada

## Abstract

**Background:**

Physicians need global health competencies to provide effective care to culturally and linguistically diverse patients. Medical schools are seeking innovative approaches to support global health learning. This pilot study evaluated e-learning versus peer-reviewed articles to improve conceptual knowledge of global health.

**Methods:**

A mixed methods study using a randomized-controlled trial (RCT) and qualitative inquiry consisting of four post-intervention focus groups. Outcomes included pre/post knowledge quiz and self-assessment measures based on validated tools from a Global Health CanMEDS Competency Model. RCT results were analyzed using SPSS-21 and focus group transcripts coded using NVivo-9 and recoded using thematic analysis.

**Results:**

One hundred and sixty-one pre-clerkship medical students from three Canadian medical schools participated in 2012–2013: 59 completed all elements of the RCT, 24 participated in the focus groups. Overall, comparing pre to post results, both groups showed a significant increase in the mean knowledge (quiz) scores and for 5/7 self-assessed competencies (*p* < 0.05). These quantitative data were triangulated with the focus groups findings that revealed knowledge acquisition with both approaches. There was no statistically significant difference between the two approaches. Participants highlighted their preference for e-learning to introduce new global health knowledge and as a repository of resources. They also mentioned personal interest in global health, online convenience and integration into the curriculum as incentives to complete the e-learning. Beta version e-learning barriers included content overload and technical difficulties.

**Conclusions:**

Both the e-learning and the peer reviewed PDF articles improved global health conceptual knowledge. Many students however, preferred e-learning given its interactive, multi-media approach, access to links and reference materials and its capacity to engage and re-engage over long periods of time.

**Electronic supplementary material:**

The online version of this article (doi:10.1186/s12909-015-0421-3) contains supplementary material, which is available to authorized users.

## Background

Global health can be defined as “an area of education, research and practice that places priority on improving health and achieving equity in health for all people worldwide” [1]. Refugee health refers to the evidence-based study of health issues related to persons forced to flee from their home countries due to conflict, torture or other forms of violence and the effect this has on their overall health. This field includes mental health, chronic and infectious conditions they are more at risk for having given their precarious migration history [2]. Refugees are an example of a vulnerable population that may benefit from physician training. Refugee health falls under the umbrella of global health.

Integrating interprofessional global health and health system training has become a priority for many medical schools in higher income countries [3]. As immigrants and refugees resettle in Western countries, physicians must enhance their knowledge and skills to meet patients’ diverse health needs [2]. The emerging fields of global and refugee health continue to captivate medical students. In response, medical schools have implemented global health curriculum [4].

E-learning is now emerging as a viable alternative to supplement and support medical student training [5]. To lay the foundation for global health, the Refugees and Global Health e-Learning Program, accessible online at http://ccirhken.ca/eLearning/, was developed and launched at the 2012 Canadian Conference for Medical Education in Banff, Alberta [6]. These online e-learning modules incorporate case studies, learning objectives, video content and global health resources. They are based on the CanMEDS roles, which is a physician competency framework describing the knowledge, skills and attitudes that physicians need to ensure better patient outcomes [7]. The CanMEDS framework is based on the seven roles that all physicians need to have (medical expert, communicator, advocate, professional, collaborator, manager and scholar) according to the Royal College of Physicians and Surgeons of Canada. The modules also make use of the Ontario Global Health Education Competencies Framework, which guides global health education programs [8] [see Additional file 1].

This pilot study sought to evaluate the effectiveness of the Refugees and Global Health e-Learning Program as a tool to introduce the basic concepts of global health to medical students and to assess the overall student experience of using the tool. Basic concepts of global health (i.e., conceptual knowledge), are essentially the first steps the learner takes on the pathway to competency in global health. Conceptual knowledge refers to essential concepts and guiding principles. Examples of some of the basic concepts students were exposed to by using the e-learning program are outlined in table form (see Discussion).

Specifically then, our first research question (RQ1) is: What is the effect of the Refugees and Global Health e-Learning Program versus peer reviewed PDF articles on the acquisition of conceptual knowledge in medical students? The second research question (RQ2) is: What was the experience for the students using the e-learning program? The authors believe that this research is timely because educators need high quality and effective resources to introduce learners to the complex field of global health.

## Methods

A sequential explanatory research design was adopted. The rationale for this approach is that the quantitative analysis provides a general understanding of the research problem and the qualitative analysis refines and explains the statistical results in more depth. This design has been discussed extensively in the mixed methods literature [9, 10].

In this design, we first collected and analyzed numeric data (phase 1). The text data were then collected, and analyzed (phase 2) and connected at the intermediate phase of the study. Phase 1 (RCT of e-learning modules versus paper reading) addressed our first research question (RQ1) and phase 2 (focus groups) addressed the second (RQ2). Thus, focusing on the same research questions and outcomes, conceptual knowledge and student experience, we used different methods to collect data and participant perspectives.

Out of the 161 medical students who completed the pre-test, 59 went on to complete all aspects of the RCT; 33 completed the e-learning program, 26 completed the PDF articles. In this paper, we will discuss the results from these 59 students. Participants were between the ages of 20 and 37 and came from a range of cultural and linguistic backgrounds; 71 % were considering a cross-cultural medical exchange, 93 % had traveled outside Canada/US, at least 66 % spoke a language other than English. Table 1 describes the characteristics of the participants.

**Table 1:** Characteristics of medical students who completed all parts of the randomized-controlled trial

Characteristic	No. (%)
Sex	
Male	21 (36 %)
Female	38 (64 %)
Perceived ethno-cultural background^a^	
White	38 (64.4 %)
Chinese	9 (15.3 %)
South Asian	8 (13.6 %)
Black	2 (3.4 %)
Korean	2 (3.4 %)
Filipino	1 (1.7 %)
Latin American	1 (1.7 %)
Arab	1 (1.7 %)
South East Asian	1 (1.7 %)
Aboriginal Peoples of North America	1 (1.7 %)
Canadian	1 (1.7 %)
Jewish	1 (1.7 %)
Persian	1 (1.7 %)
Ukrainian	1 (1.7 %)
Language spoken	
English	59 (100 %)
French	21 (35.6 %)
Other	39 (66 %)
Previously traveled outside Canada & US	55 (93 %)
Previously volunteered with marginalized or disadvantaged populations	41 (70 %)
Previously completed a Global Health Learning elective	6 (10 %)
Considering a clinical rotation outside of the country	42 (71 %)

^a^Some students perceived they were from more than one cultural background

This study was approved by the Ottawa Hospital Research Ethics Boards and the Bruyère Continuing Care Research Ethics Board for all institutions as well as the Health Research Ethics Authority in the Province of Newfoundland and Labrador.

### Participants

Three Canadian medical schools participated in this study: University of Ottawa, Memorial University of Newfoundland and University of Saskatchewan. One hundred and sixty one pre-clerkship medical students at the three sites were invited to participate in the study by email invitation early in the 2012–2013 school year.

### Data collection

#### Phase 1: randomized-controlled trial (RQ1)

In the initial e-mail invitation, students were asked to complete an online knowledge quiz and a validated global health self-assessment questionnaire [11] [see Additional file 2] on SurveyMonkey™. Students who completed this first step were randomly assigned a link to access either a) the closed beta-version of the Refugees and Global Health e-Learning Program, or b) two global health education articles in PDF format on the definitions for global health [1] and on ethics and best practice guidelines for global health training [12]. The intervention group was assigned the e-learning modules. Each group completed the same pre/post knowledge quiz and questionnaire before and after the intervention.

##### Self-assessment questionnaire (RQ1)

The CanMEDS for Global Health Self-Assessment Tool [13], accessible at the following link: http://www.ruor.uottawa.ca/handle/10393/23275 was adapted to assess students’ self-perceived awareness of global health concepts. The self-assessment questionnaire is composed of 22 items and uses a seven point Likert scale ranging from strongly disagree to strongly agree. It was used to measure the degree of change in students’ self-perceived knowledge pre and post intervention.

##### Knowledge Quiz (RQ1)

The knowledge quiz contains 30 multiple choice questions based on the core CanMEDS competencies covering fundamental global and refugee health material. This knowledge quiz was developed and refined by global health experts and pilot-tested with 26 pre-clinical medical students during a national summer institute on refugee health in Ottawa, Canada. Once the study was completed, students were provided access to all of the education materials.

#### Phase 2: focus groups (RQ2)

After the results from the self-assessment questionnaire and the knowledge quiz were analyzed, those deemed by the researchers as significant or interesting were used to create focus group protocol questions [see Additional file 3]. For example, the quantitative results showed both learning approaches were effective for knowledge acquisition, but did students prefer one approach over the other? Focus group questions were created to explore students’ personal experiences and perceptions of the e-learning program. Students who fully participated in the RCT were invited to take part in focus groups. Altogether, 24 students participated in the four focus group sessions; five to seven participants in each session, with 14 doing the e-learning modules (intervention) and 10 doing the PDF articles (control). The sessions were conducted in a semi-structured and open-ended manner by moderators with knowledge in refugee and global health. All focus group interviews were audio taped and transcribed verbatim. The interview guide studied the e-learning program’s features, its barriers and its facilitators, the learning outcomes including CanMEDS conceptual knowledge, future career goal changes and the possibility of integrating the program into the curriculum.

### Data analysis

#### Quantitative data analysis (RQ1)

The quantitative data from the knowledge quiz and self-assessment questionnaire were analyzed using the Statistical Package for Social Sciences (SPSS) version 21 (SPSS Inc., Chicago, IL). Descriptive statistics, and repeated measures t-tests were performed to determine any statistically significant difference between subgroups of students.

#### Qualitative data analysis (RQ2)

Five researchers from our team (D.G., J.A., I.B., A.M., M.B.) independently reviewed the transcribed interviews from the focus groups using thematic analysis. After an initial exploratory analysis, the researchers began the coding process. The codes were discussed and debated by the researchers in order to narrow the codes into a few themes. Themes were then edited to determine common ones. Outliers were sought and explanations considered. We then used NVivo 9 qualitative data analysis software (QSR International Pty Ltd., Doncaster, Victoria, Australia) to group the themes more specifically and the team met again to review and ultimately agree on the main themes (interpretations) and key sub themes. To ensure reliability, member checking was done at each institution having focus groups participants comment on emerging themes in comparison with their experience.

## Results

### Quantitative results

#### Self-assessment questionnaire (RQ1)

After conducting a paired samples *t*-test for both the control and intervention groups, there was a statistically significant difference between the pre and post-test results for the self-perceived competency questionnaire in the following CanMEDS roles: health advocate, medical professional, scholar, manager, and medical expert (see Table 2). Overall there was an increase in conceptual knowledge in both groups with a change from the pre-test (M = 23.19/30, SD = 2.87) to the post-test (M = 24.15/30, SD = 2.94), t(58) = −2.36, *p* < 0.05 (two-tailed). The mean increase in scores was 0.97 with a 95 % confidence interval ranging from −1.79 to −0.15. The standard effect sizes for the mean differences of each role were calculated using Cohen’s *d* (post-score minus pre-score, divided by the standard deviation of pre and post-scores combined) [14]. We found the effect sizes for all roles were small. Furthermore, we found no statistically significant differences between those students who have travelled outside of North America or participated in cross cultural medical experiences.

**Table 2:** Changes in pre-test and post-test scores of the self-assessment questionnaires grouped under the CanMEDS roles

Role	Pre-test		Post-test		df	t	Cohen’s *d*	Difference
	Mean score	SD	Mean score	SD				
Communicator	4.82	.65	4.97	.68	58	−1.53	0.20	Small
Collaborator	6.19	.78	6.31	.74	58	−.85	0.11	Small
Health advocate	4.84	.76	5.21	.74	56	−3.35*	0.44	Small
Medical professional	5.14	.70	5.42	.68	57	−2.94*	0.41	Small
Scholar	4.60	1.38	4.85	1.19	58	−1.87*	0.24	Small
Manager	4.76	1.13	5.29	1.03	57	−3.18*	0.44	Small
Medical expert	5.70	1.02	6.08	.70	58	−3.16*	0.41	Small

Standardized Effect Sizes, Cohen’s *d*, = post-score minus pre-score, divided by the standard deviation of pre and post-scores (combined). Qualitative differences: “Large” = values of ≥0.8; “Moderate” = values between 0.50 and 0.79; “Small” = values below 0.50

*p < .05

#### Knowledge quiz results (RQ1)

There was no difference in mean scores on the CanMEDS constructs of the quiz between those who completed the PDF articles and those who completed the e-learning modules (see Table 3). Statistical power in this pilot study was not sufficient to detect small differences between the two learning approaches. Furthermore, there were no significant differences between the learning medium and demographic information collected on the quiz such as gender and country of birth. Content evidence for the quiz was established by verifying the quiz questions and responses by numerous experts in the field including the authors and the undergraduate global health director. A reliability analysis was conducted for the items under each of the CanMEDS roles. However, as there were few items below each role and only 59 students, the knowledge quiz showed poor internal consistency; Cronbach’s alpha coefficients ranged from .027 for the Communicator items to .458 for the Collaborator items.

**Table 3:** Differences in mean quiz scores between students who completed the PDF articles or the e-learning modules

	PDF articles		e-Learning modules		df	t
	Mean score	SD	Mean score	SD		
Pre-test	22.91/30	3.24	23.60/30	2.36	56	−.90
Post-test	23.76/30	2.14	24.72/30	3.76	56	−.123

### Qualitative results (RQ2)

Analysis of the focus group transcripts revealed no systematic differences between the different institutions; therefore we combined the codes of the four groups. We identified three broad themes regarding the use of e-learning modules as a tool to introduce the CanMEDS global health concepts (see Table 4), designated as: (1) facilitators, (2) barriers, (3) curriculum delivery using e-learning.

**Table 4:** Summary of themes identified through the analysis of focus group transcripts

Facilitators	Barriers	Curriculum delivery using e-learning
• Learning style	• Learning style	• Introduction to the basic global health concepts
• Personal interest in global health	• Work overload	• Conceptual knowledge acquisition of the CanMEDS’ competencies
• Ease of access	• Time constraint	• Factual learning
• Flexibility and convenience	• Technical problems	• Independent learning
• Interactive content	• Lack of face to face interactions	• Combination of teaching methods
• Knowledge assessment	• Potential for distraction (ie. other links)	• Potential improvements
• Applicability and relevance of the e-Learning Program		
• Integration into the official curriculum		

#### Category 1: facilitators (RQ2)

Medical students identified a variety of facilitators which aided in completing the e-learning modules. The most prevalent reasons for completing the e-learning modules, as expressed in the focus groups, were pertaining to the medical students’ “learning style” preference and their personal interest towards global health, as illustrated in the following comment: “I think it all depends on how interested you are and how relevant you see it as. Even if you are super busy you make time for things that are important to you and things you think are going to help you as a doctor and/or just as a person.”

On another note, students referred to the many facilitators related to the e-learning program itself. Comments revolved around the “ease of access,” “flexibility and convenience,” and “interactive content.” In the words of one student, “[there] were also a lot of interactive things that e-learning offer that you can’t give in a lecture.”

Another sub-theme related to the relevance of the e-learning program itself. Students seemed more eager to complete the e-learning modules if these were regarded as relevant for the current curriculum or for future practice. As one participant stated, “[in] any field of medicine you will probably end up seeing patients coming in, or you may be collaborating with different professionals who are from different cultural backgrounds.”

Taking this further, integration into the official curriculum as a mandatory course with designated allotted time would clearly be a motivator as one student commented, “I think it is important to consider the time you spend on the e-learning as part of class time so that you don’t overwhelm students.”

Students also felt it was important that these global health concepts related to the actual CanMEDS competencies being taught in the curriculum, as one student states, “[part] of me getting into the global health learning was the CanMEDS and learning more about the CanMEDS so if you can make that link that might get students to do the e-learning.”

#### Category 2: barriers (RQ2)

Students also identified barriers to the completion of the e-learning program. These barriers have been classified in 2 categories: 1) the barriers associated with student workload and 2) the barriers related to the e-learning modules.

Regarding the workload barriers, focus group participants mentioned a number of issues including different learning styles, and “work overload.” The issue of work overload was illustrated by the comment, “[having] to go home and not only complete school work but then go online and read at the computer which you don’t want to do after a long day, it’s tough.”

Barriers associated with the e-learning modules themselves included predominantly “technical problems,” as a student noted, “I think technical glitches are one of the biggest frustrations with e-learning courses. When they work really well they are fabulous but if things start to glitch I am probably going to get really frustrated.”

Students were also concerned about the “lack of interaction” and the “lack of flexibility,” both of which are voiced in the following comment, “[because] if you run into something [in an] e-learning session that doesn’t make sense then you do not have any opportunity to ask for clarification so it is very structured and very linear. You can move backwards and forwards but there is a path that you are following and you cannot deviate from it.”

Various students complained about the overwhelming content of the modules, as illustrated by the following comment, “I did find it a bit wordy. I thought there was a lot of content and I suppose each module seemed to take a bit longer than I expected to get through so I was left wondering *how much is left?*.”

#### Category 3: curriculum delivery using e-Learning (RQ2)

Students were also asked to discuss the effectiveness of the Refugees and Global Health e-Learning Program and its potential integration into the current curriculum. Student opinions seemed split. Many participants indicated the e-learning modules could be used as a tool to introduce basic concepts, and provide an opportunity for those more interested to further their knowledge. This is captured by the following comment, “[it] was actually a good place to start because it also gave you references and supplemental videos to watch.”

Students also reported that the e-learning modules improved their understanding (conceptual knowledge) of the complexity and challenges of communication and collaboration in global health settings. For example, “[it] really helped me for how I would be prepared to talk to a refugee” and “I gained knowledge on the medical expert [role], just because the module was more based on treating the person as a whole and not really their illness or disease.”

Students were also in favour of a “combination of teaching methods” and using the e-learning program as a supplement to the lectures. In fact, all mentioned the importance of face to face interactions with small group discussions being particularly effective as a learning method. This is illustrated in the following comments, “[it] could be used beneficially if maybe they [modules] were viewed as supplement to the lecture.”

Finally, students gave valuable insights in terms of potential improvements to the delivery of the content of this e-learning program. Many felt the need to have a clearer outline with progression markers. As one student noted, “[little] bars that show you your progress on modules are really useful.”

Students also highlighted the usefulness of a resource person as a way to overcome the lack of interactivity during the completion of the e-learning program. For example one student commented, “[maybe] one person that you could contact with questions.”

## Discussion

Medical schools continue to work toward the integration of global health content into their curricula. At the same time evidence is mounting on the benefits of e-learning, which is equivalent to, and in some cases better than, traditional textbook learning in enabling knowledge acquisition [15]. Moreover, the literature suggests higher student satisfaction rates with e-learning, particularly given its convenience, flexibility and interactivity [5]. Our evaluation of the integration of global and refugee health e-learning is consistent with this literature [16].

The RCT demonstrated that both the e-learning program and the PDF articles yielded a significant improvement in global health conceptual knowledge and self-perceived competency, without any significant difference between both interventions. Although the RCT failed to show a significant difference between the e-learning modules and the peer reviewed articles (RQ1), there was general consensus from the focus groups that the e-learning modules were preferred by the majority of medical students (RQ2) and thus an effective tool to deliver global health conceptual knowledge [17].

The qualitative results demonstrated the students’ preference for the e-learning program as a tool to deliver curriculum citing enhanced convenience, flexibility, interactive content and ease of access [18]. E-learning was regarded as engaging by facilitating independent learning and as an excellent introductory tool for global health. It could be used in combination with other teaching methods and as a valuable reference tool for the future.

As with any educational method, there were perceived limitations. Students had different learning preferences, and some were concerned about the lack of face to face interactions in e-learning. Others offered ways to improve the presentation of content, such as including a simpler outline and the need for progression markers (i.e., page numbers), to help with time management. The modules have all been modified to take into account many of these delivery and integration concerns. Despite ongoing editing there may remain some technical challenges such as technical problems with internet, computer glitches and external internet links that can distract the students [19].

The quantitative findings highlighted an improvement in all CanMEDS global health concepts (see Table 5), both perceived (questionnaire) and knowledge based (quiz), with the notable exception of the roles of communicator and collaborator. This absence of significant improvement in the collaborator role may be attributable to a ceiling effect, given that the baseline mean score was 6.19/7, i.e., there was not much room for improvement. Yet it is significant to note that the focus groups emphasized the usefulness of the e-learning program specifically to develop conceptual knowledge particularly for the role of communicator and also for the role of collaborator. This finding is intriguing and reinforces the value of mixing qualitative research when assessing learning outcomes [5, 20].

**Table 5:** Basic global health concepts students were exposed to by using the e-learning program or reading the articles

CanMEDS roles	Global health concepts
Expert	Demonstrate an awareness of how war, conflict, and famine impact the health of individuals.
Communicator	Recognize how your own cultural biases, values and belief systems may affect your interaction with patients.
Collaborator	Skills include assessing problems, identifying key players, listening to team members, and working together in design and implementation of programs.
Manager	In humanitarian contexts, manager skills play a critical role in directing human resources, engaging and training local staff, networking with nongovernmental organizations, and effectively utilizing limited resources.
Advocate	Being a health advocate means treating your patient in their own particular context, without dismissing their cultural concerns.
Scholar	As scholars, professionals demonstrate a lifelong commitment to learning, as well as the creation of knowledge.
Professional	Professionals learn to maintain healthy boundaries to keep both themselves and their patients safe.

### Strengths and limitations

There were several limitations to this pilot study. We did not enroll as many students as we anticipated. We felt this was due to our choice not to use incentives and our inability to properly introduce the study to students due to university rules. In addition, our comparison intervention contained similar content to the e-learning modules. We learned from the primary outcome of this study that to detect knowledge differences in our education approaches would require much more power, in the magnitude of 400+ participants per group for greater precision and reliability. This voluntary learning project may have attracted students with an interest in global health and while we feel this may not have reduced knowledge acquisition, contamination between learning materials may have reduced the probability of finding a difference in knowledge acquisition between the similar learning approaches. For example, 71 % of participants were considering a cross-cultural medical elective and may have already read the PDF articles.

Other sources of global health material in the curriculum may have confounded our results and affected the overall outcome difference between the two groups. As for the self-assessment tool, it was limited by a potentially variable baseline of self-assessments that would then make changes more difficult to interpret. Finally, we used a beta-version of the e-learning program which may not have been as effective as the final e-learning which improved as a result of students’ comments in this study.

These limitations were mitigated at various levels through the methodology. The single blinded RCT helped to control for bias. In addition, the diversity of the participants coming from multiple universities reduced selection bias. The validity of the qualitative data was ensured by several means, including maximum variation sampling, having several coders, member-checking and negative case analysis. In addition, the mixed-methods design itself can be regarded as a strength since both quantitative and qualitative methods together provide more evidence for studying a research problem than either method alone [10]. The RCT provided rigor to this study and enabled an objective comparison of outcomes following the use of the e-learning program and the PDF articles. The quantitative aspect alone wouldn’t have allowed for an accurate evaluation of the impact of the e-learning program on medical students. In fact, the qualitative data were fundamental for an authentic appraisal of the e-learning as a learning method and a thorough inquiry about the students’ conceptual knowledge acquisition following the use of the e-learning program.

## Conclusion

Global health and social accountability are being introduced into the medical school curriculum with varying degrees of success. Introductory global health curriculum, before students begin their field work, often does not happen. The PDF files, and the e-learning modules were effective approaches as both showed improved global health knowledge scores, but no statistically significant difference was found between the two. More research is needed to refine the effectiveness and support the integration of such introductory efforts in the curriculum.

With the support of students and educators at collaborating universities, the Refugees and Global Health e-Learning Program is now being used as part of the introductory global health curriculum for community service learning field work and it could also potentially be used to supplement current orientation provided to students prior to their global health experience. A significant proportion of the students preferred the e-learning due to its convenience, flexibility and interactivity. This would suggest acceptability of the e-learning program. The e-learning program was described as engaging and informative, but will likely be best introduced and utilized in conjunction with other face to face teaching, such as small group sessions with a global health expert. In certain institutions where there are no global health experts available, this e-learning could potentially provide a primer for the learner. More study is needed to assess whether an early learners exposure to and acquisition of global health conceptual knowledge translates into social accountability downstream in one’s future career.

## Additional files

Additional file 1: **Framework for Global Health Education in Postgraduate Family Medicine Training (CanMEDS competencies).** (PDF 195 kb) Additional file 2: **Validated Global Health Self-Assessment Questionnaire and Knowledge Quiz (questions used for the first and last step of the randomized-controlled trial).** (PDF 742 kb) Additional file 3: **Focus groups questions (questions used for the qualitative component of the study).** (PDF 103 kb)

## Abbreviations

RCT: Randomized-controlled trial
SPSS: Statistical package for social sciences
