# Supplementary material for: Introducing global health into the undergraduate medical school curriculum using an e-learning program: a mixed method pilot study
Source: BMC Med Educ. 2015 Sep 2;15:142. doi: 10.1186/s12909-015-0421-3 (PMC4557599; doi:10.1186/s12909-015-0421-3)
Supplement: Additional file 1: — Framework for Global Health Education in Postgraduate Family Medicine Training (CanMEDS competencies). (PDF 195 kb) [file 12909_2015_421_MOESM1_ESM.pdf]

Additional file 1: Framework for Global Health Education in Postgraduate Family Medicine Training

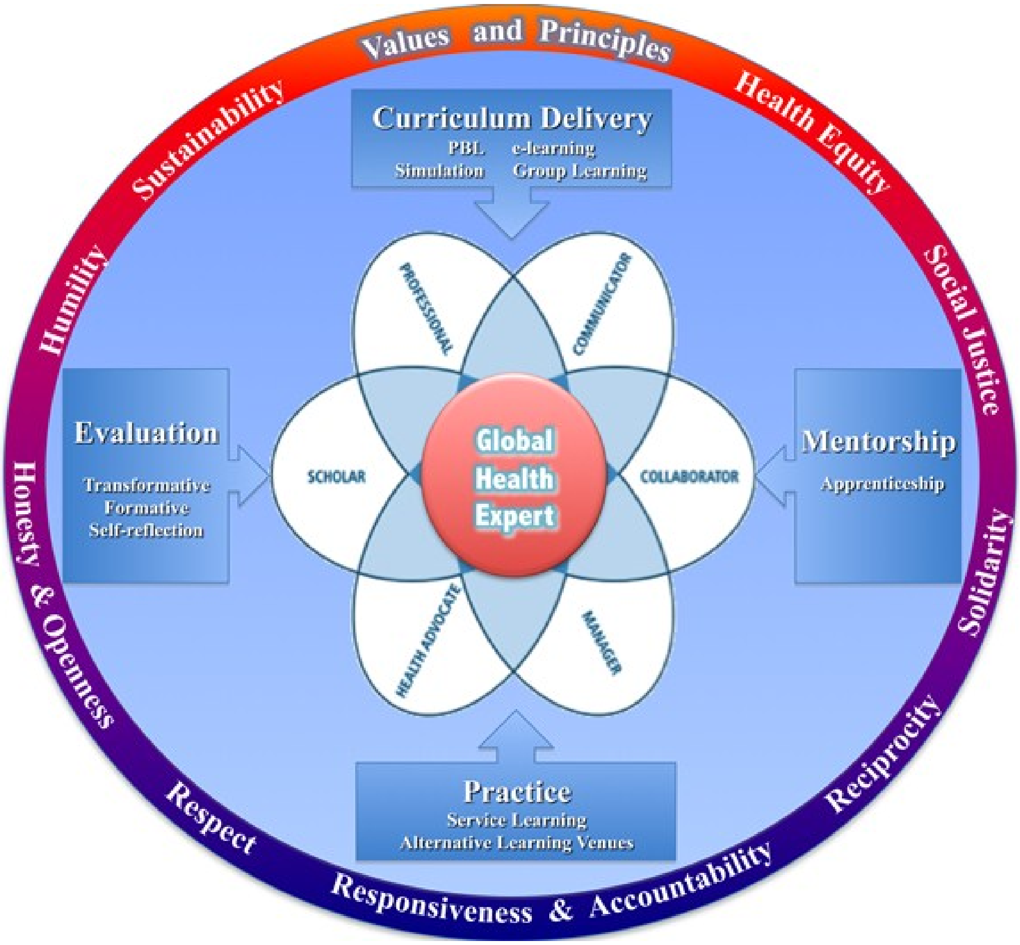

© 2010, Ontario Family Medicine Global Health Education Working Group

*Permission has been provided by the authors Dr. Redwood-Campbell and Dr. Rouleau to reprint this figure in this publication.
