# Supplementary material for: Introducing global health into the undergraduate medical school curriculum using an e-learning program: a mixed method pilot study
Source: BMC Med Educ. 2015 Sep 2;15:142. doi: 10.1186/s12909-015-0421-3 (PMC4557599; doi:10.1186/s12909-015-0421-3)
Supplement: Additional file 2: — Validated Global Health Self-Assessment Questionnaire and Knowledge Quiz (questions used for the first and last step of the randomized-controlled trial). (PDF 742 kb) [file 12909_2015_421_MOESM2_ESM.pdf]

Additional file 2: Validated Global Health Self-Assessment Questionnaire and Knowledge Quiz

uOttawa

uOttawa Summer Institute in Refugee Health

a) **For your unique participant code, please provide your mother's first name initial, the day and month of her birthday (e.g. You should enter H3006 for Helen born on June 30th)**

   ____

b) **Which learning assignment have you completed?**

   ☐ E-Learning Module

   ☐ Articles (PDFs)

   ☐ N/A

PARTICIPANT DEMOGRAPHICS: (Please complete)

1. **In what year were you born?**

19__

2. **What is your gender?**

☐ Female ☐Male

3. **In what country were you born?**

____

4. **Please indicate from the list below what you identify as your ethnic background (mark all that apply).**

☐ White

☐ Chinese

☐ South Asian (e.g. East Indian, Pakistani, Sri Lankan, etc.)

☐ Japanese

☐ Southeast Asian (e.g. Cambodian, Indonesian, Laotian, Vietnamese, etc.)

☐ West Asian (e.g., Afghan, Iranian, etc.)

☐ Korean

☐ Black

☐ Filipino

☐ Latin American

☐ Arab

☐ Aboriginal Peoples of North America (North American Indian, Métis, Inuit)

☐ Other, please specify: ____________________

5. What languages are you able to speak?

☐ English

☐ French

☐ Other: specify _______________________

6. How would you rate your English language proficiency?

☐ Excellent

☐ Very Good

☐ Good

☐ Poor

7. Have you traveled outside of Canada and the United States?

☐ Yes ☐ No

If Yes; Where? and When?

____

8. Have you ever volunteered to work with marginalized or disadvantaged populations?

☐ Yes ☐ No

If Yes; Which populations? ___________________

9. **It is early in your career as a healthcare professional, however please indicate if you envision pursuing a career in:**

☐ Family medicine

☐ Another specialty (e.g. surgery, cardiology etc.)

☐ Do not know

10. **In the future do you plan to participate in clinical rotation(s) outside the country where you study?**

☐ Yes ☐ No

11. **Have you completed a Refugee or Global Health Elective?**

☐ Yes ☐ No

If Yes, Where? _____________

12. **Have you completed any Refugee or Global Health E-Learning Programs?**

☐ Yes ☐ No

If Yes; Which program? __________________

GLOBAL HEALTH SELF-PRECEIVED COMPETENCY QUESTIONNAIRE

Instructions: Each section below identifies a different role that practitioners play as a health professional; for example, a health professional needs to be a good communicator with her patients and an effective collaborator with other professionals.

Envision yourself within these roles and carefully read and rate the extent to which you agree with each statement. The options range from strongly disagree to strongly agree as seen below. Please mark only one option per question.

☐ Strongly Disagree	☐ Disagree	☐ Somewhat Disagree	☐ Neutral	☐ Somewhat Agree	☐ Agree	☐ Strongly Agree	☐ Not Applicable

This questionnaire uses the term "marginalized patients/people". We define a marginalized person as someone who may face health services, prevention and treatment barriers.

As a communicator:

1. I am comfortable building open and trusting relationships with marginalized patients/people.

☐ Strongly Disagree	☐ Disagree	☐ Somewhat Disagree	☐ Neutral	☐ Somewhat Agree	☐ Agree	☐ Strongly Agree	☐ Not Applicable

2. I feel it will be a challenge to listen actively to concerns of patients/people who are marginalized.

☐ Strongly Disagree	☐ Disagree	☐ Somewhat Disagree	☐ Neutral	☐ Somewhat Agree	☐ Agree	☐ Strongly Agree	☐ Not Applicable

3. I am confident that I can express my ideas and concerns clearly to patients/people who are marginalized.

☐ Strongly Disagree	☐ Disagree	☐ Somewhat Disagree	☐ Neutral	☐ Somewhat Agree	☐ Agree	☐ Strongly Agree	☐ Not Applicable

As a collaborator:

4. I understand the importance of building partnerships with medical and other health professionals to benefit patients who are marginalized.

☐ Strongly Disagree	☐ Disagree	☐ Somewhat Disagree	☐ Neutral	☐ Somewhat Agree	☐ Agree	☐ Strongly Agree	☐ Not Applicable

5. I understand the role of nurses, pharmacists, social workers and other allied health professionals when working with patients who are marginalized.

☐ Strongly Disagree	☐ Disagree	☐ Somewhat Disagree	☐ Neutral	☐ Somewhat Agree	☐ Agree	☐ Strongly Agree	☐ Not Applicable

As an advocate (for patients):

6. I am aware of the obstacles faced by marginalized people in seeking access to health care.

☐ Strongly Disagree	☐ Disagree	☐ Somewhat Disagree	☐ Neutral	☐ Somewhat Agree	☐ Agree	☐ Strongly Agree	☐ Not Applicable

7. I have an accurate understanding of the relationship between income and health outcomes.

☐ Strongly Disagree	☐ Disagree	☐ Somewhat Disagree	☐ Neutral	☐ Somewhat Agree	☐ Agree	☐ Strongly Agree	☐ Not Applicable

8. I think it will be challenging to identify the needs of patients who are marginalized.

☐ Strongly Disagree	☐ Disagree	☐ Somewhat Disagree	☐ Neutral	☐ Somewhat Agree	☐ Agree	☐ Strongly Agree	☐ Not Applicable

9. I am aware of the medical services available to patients who are marginalized.

☐ Strongly Disagree	☐ Disagree	☐ Somewhat Disagree	☐ Neutral	☐ Somewhat Agree	☐ Agree	☐ Strongly Agree	☐ Not Applicable

As a medical professional:

10. I feel it will be challenging to provide medical care to my patients who are marginalized.

☐ Strongly Disagree	☐ Disagree	☐ Somewhat Disagree	☐ Neutral	☐ Somewhat Agree	☐ Agree	☐ Strongly Agree	☐ Not Applicable

11. I am confident that I will attempt to understand perspectives of my patients who are marginalized.

☐ Strongly Disagree	☐ Disagree	☐ Somewhat Disagree	☐ Neutral	☐ Somewhat Agree	☐ Agree	☐ Strongly Agree	☐ Not Applicable

12. I feel it will be challenging to discuss sensitive issues (e.g. alcohol, drugs, sexual issues) with my patients who are marginalized.

☐ Strongly Disagree	☐ Disagree	☐ Somewhat Disagree	☐ Neutral	☐ Somewhat Agree	☐ Agree	☐ Strongly Agree	☐ Not Applicable

13. When interacting with marginalized patients, I will be aware of my own limitations.

☐ Strongly Disagree	☐ Disagree	☐ Somewhat Disagree	☐ Neutral	☐ Somewhat Agree	☐ Agree	☐ Strongly Agree	☐ Not Applicable

As a scholar:

14. I actively participate in global health activities.

☐ Strongly Disagree	☐ Disagree	☐ Somewhat Disagree	☐ Neutral	☐ Somewhat Agree	☐ Agree	☐ Strongly Agree	☐ Not Applicable

15. I know how to access resources to keep up to date with global health issues.

☐ Strongly Disagree	☐ Disagree	☐ Somewhat Disagree	☐ Neutral	☐ Somewhat Agree	☐ Agree	☐ Strongly Agree	☐ Not Applicable

16. I have read journal articles, books, or other educational materials on sociocultural aspects of health.

☐ Strongly Disagree	☐ Disagree	☐ Somewhat Disagree	☐ Neutral	☐ Somewhat Agree	☐ Agree	☐ Strongly Agree	☐ Not Applicable

As a manager:

17. I am aware of the challenges in providing good quality care within the available time of a patient visit.

☐ Strongly Disagree	☐ Disagree	☐ Somewhat Disagree	☐ Neutral	☐ Somewhat Agree	☐ Agree	☐ Strongly Agree	☐ Not Applicable

18. I know how to use the expertise of nurses and other health professionals so that my time is used more efficiently.

☐ Strongly Disagree	☐ Disagree	☐ Somewhat Disagree	☐ Neutral	☐ Somewhat Agree	☐ Agree	☐ Strongly Agree	☐ Not Applicable

As an expert (knowledge):

19. I understand the relationship between health and the social determinants of health and how these social determinants vary across world regions.

☐ Strongly Disagree	☐ Disagree	☐ Somewhat Disagree	☐ Neutral	☐ Somewhat Agree	☐ Agree	☐ Strongly Agree	☐ Not Applicable

20. I understand how cultural background, socioeconomic status and language barriers can influence access to care and health outcomes.

☐ Strongly Disagree	☐ Disagree	☐ Somewhat Disagree	☐ Neutral	☐ Somewhat Agree	☐ Agree	☐ Strongly Agree	☐ Not Applicable

21. I have a good understanding of how access to clean water, sanitation and nutrition affect individuals as well as population health.

☐ Strongly Disagree	☐ Disagree	☐ Somewhat Disagree	☐ Neutral	☐ Somewhat Agree	☐ Agree	☐ Strongly Agree	☐ Not Applicable

Global Health Competencies:

22. Do you feel that you may have initially overestimated your global health competencies?

☐ Yes	☐ Maybe	☐ No	☐ Undecided

Comments:

Thank you very much for completing this global health competency questionnaire.

uOttawa

uOttawa Summer Institute in Refugee Health

Refugee and Global Health e-Learning Quiz

Please check **one** answer for each question.

	Question	Answer
**A**	**INTRODUCTION**	
**1.**	**Which of the following principles and values is most important for global health?**	
a)	Humility	☐
b)	Sustainability	☐
c)	Equity	☐
d)	All principles and values are equally important.	☐
**2.**	**Which of the following is NOT one of the 7 CanMed core role competencies?**	
a)	Manager	☐
b)	Advocate	☐
c)	Mentor	☐
d)	Scholar	☐
**3.**	**What is essential for a career in global health?**	
a)	Service learning abroad	☐
b)	Global health knowledge, skills and values	☐
c)	Ability to speak more than one language	☐

d)	Medical degree	☐
**4.**	**How many people around the world currently are uprooted due to war?**	
a)	32 million	☐
b)	37 million	☐
c)	42 million	☐
d)	52 million	☐
**B**	**COMMUNICATOR**	
**5.**	**Based on evidence from patients who cannot communicate effectively with their doctors due to a language barrier, which of the following demonstrate(s) the need for medical interpretation?**	
a)	Patients are less satisfied with care received.	☐
b)	Patients seek less care in the Emergency Department.	☐
c)	Patients have a higher chance of being misdiagnosed and/or prescribed inappropriate medication.	☐
d)	A & C	☐
**6.**	**Which of the following types of interpreter is characterised by the ability to provide cultural interpretation, assures high confidentiality, and requires booking and coordination?**	
a)	Telephone interpretation service	☐
b)	Family member or friend	☐
c)	Professionally trained medical/cultural interpreter	☐
d)	Ad-hoc interpreter	☐
**7.**	**Which one of the following is true in terms of family members acting as interpreters?**	
a)	Family members can assure confidentiality.	☐
b)	Family members can effectively address sensitive subjects.	☐

c)	Family members sometimes withhold important information.	☐
d)	Family members understand the medical conditions of their loved ones.	☐
**8.**	**During the patient/doctor interview, which of the following should be followed?**	
a)	Triangle seating arrangement is preferred to improve the physician-patient connection.	☐
b)	Interpreter should sit next to the patients or slightly behind to improve the physician-patient connection.	☐
c)	To save time, there is no need for lengthy introductions.	☐
d)	All of the above	☐
**9.**	**Which of the following is <u>not</u> a step to be taken at the <u>end</u> of the interview session?**	
a)	Explain the interpreter's role to the patient.	☐
b)	Repeat important concepts.	☐
c)	Review treatment plan carefully.	☐
d)	Encourage patients to ask questions.	☐
**C**	**MEDICAL EXPERT**	
**10.**	**The role of the medical expert includes:**	
a)	An awareness of how war, conflict and famine impact the health of individuals.	☐
b)	Having an understanding of the barriers refugees face when trying to access health care.	☐
c)	The ability to discover the challenges of treating and preventing disease in resource-limited settings.	☐
d)	All of the above	☐
**11.**	**The key to treating refugee health problems is:**	
a)	A thorough understanding of the pathophysiology of tropical diseases.	☐
b)	Maintaining a reliable supply of electricity in the clinic.	☐
c)	Disease prevention strategies and understanding the context of the situations patients are coming from.	☐

d)	Having samples of antibiotics to give to patients.	☐
**12.**	**Which of the following is/are barrier(s) refugees face when accessing the Canadian health care system?**	
a)	The provider's beliefs, knowledge, cultural sensitivity and cultural competence	☐
b)	Cost of interpretation services	☐
c)	Education level of the refugees	☐
d)	All of the above	☐
**13.**	**Refugees may present with a wide range of illnesses. Medical experts need to:**	
a)	Be aware of global patterns of burden of disease.	☐
b)	Screen refugees newly arriving from South Asia and Africa for *Schistosoma.*	☐
c)	Obtain a detailed history of the entire trauma a patient has gone through.	☐
d)	All of the above	☐
**14.**	**When working in a resource-limited setting either here in Canada (northern/remote) or overseas, medical experts need to:**	
a)	Practice medicine that is traditional to the area.	☐
b)	Be prepared to work with traditional healers to provide the best possible care.	☐
c)	Perform the cultural practices expected by patients.	☐
d)	Ignore all cultural and traditional practices as they are inferior to western medicine.	☐
**D**	**ADVOCATE**	
**15.**	**Which of the following are needed for health advocacy?**	
a)	Adaptability	☐
b)	Mobility	☐
c)	Neutrality	☐
d)	All of the above	☐

**16.**	**Which of the following is particularly important in the role of a health advocate?**	
a)	Effective verbal and non verbal communication.	☐
b)	A health practitioner's responsibility goes beyond the care of the individual patient.	☐
c)	Professional competence and lifelong learning.	☐
d)	Formal training in International Relations.	☐
**17.**	**'Developing and maintaining networks with other social activists and marginalised patients across work settings is one way to prevent burnout". At which career stage is the risk of burnout particularly high?**	
a)	Early-career in which student projects generally have little continuity.	☐
b)	Mid-career where successive placements are often highly intense.	☐
c)	Established-career when they are often effective advocates and a motivation to others.	☐
d)	Retirement when there is a possibility of not having a sense of purpose anymore.	☐
**E**	**PROFESSIONAL**	
**18.**	**What is professionalism in the global health context?**	
a)	Being well dressed.	☐
b)	Playing the role of "medical ambassador" and acting within one's knowledge and skill level in all work settings.	☐
c)	Fulfilling clearly defined roles and responsibilities in a familiar setting.	☐
d)	None of the above	☐
**19.**	**What does ethical engagement lead to?**	
a)	Safety	☐
b)	Quality care	☐
c)	Sustainability	☐
d)	All of the above	☐
**20.**	**What activities should be done prior to embarking on a global health elective? Choose the best answer.**	

a)	Visit a travel clinic to ensure all your vaccinations are up to date.	☐
b)	Act ethically when engaging in medical practice including self-reflection, journaling and discussing issues with peers and mentors.	☐
c)	Participate in pre departure training including topics such as health and safety, cultural and language competency and ethical considerations.	☐
d)	De-brief with other students or mentors and plan to better prepare for subsequent trips.	☐
**21.**	**You are working within a resource-limited setting in Canada or overseas. A supervisor asks you to perform a task that you know you are not qualified to do. How should you proceed?**	
a)	Go with your gut feeling.	☐
b)	Follow your supervisor's directions.	☐
c)	Reflect on the ethical implications of your actions and practice within your knowledge and skills level.	☐
d)	Do whatever benefits your personal learning the most.	☐
**F**	**COLLABORATOR**	
**22.**	**Refugee health networks are collaborative structures with the aim to improve access for refugees to the healthcare system. When creating such a network, what would you do first?**	
a)	Create the governance structure.	☐
b)	Engage recent/past refugees.	☐
c)	Investigate best practices.	☐
d)	Find locations where clinics can be set up.	☐
**23.**	**Which of the following statements accurately describe(s) collaboration to improve health care delivery?**	
a)	Evidence indicates that lack of communication and collaboration between healthcare providers can seriously harm patients.	☐
b)	Collaboration requires the capacity to adjust with different approaches and skills to work in multi-cultural settings to ensure optimum care of vulnerable populations.	☐
c)	Interdisciplinary teams provide a diverse approach to care, which is helpful in chronic	☐

	disease management.	
d)	All of the above	☐
**24.**	**In order to effectively manage collaborative teams, individuals need to:**	
a)	Respect difference and become skilful in managing errors and misunderstandings with others to maintain positive working environments.	☐
b)	Possess essential skills in being able to assess problems, identify key players, listen to team members, and work together to design and implement programs.	☐
c)	Have the physician take the lead in discussions.	☐
d)	A & B	☐
**G**	**MANAGER**	
**25.**	**The role of the manager in the healthcare setting is:**	
a)	To obtain research funding.	☐
b)	To deal with all administrative duties.	☐
c)	To ensure the clinic is able to deal with complex situations and provide quality care in resource-limited settings.	☐
d)	To act as a locum physician when needed.	☐
**26.**	**Some of the most vulnerable refugees are (select the best answer):**	
a)	Urban refugees in an urban environment.	☐
b)	Women and children.	☐
c)	Male-headed households.	☐
d)	Refugees resettled with their family.	☐
**27.**	**Long-term goals of the manager include:**	
a)	Limiting unnecessary referrals to Emergency Departments.	☐
b)	Integrating refugees into the Canadian healthcare system.	☐
c)	Identifying disease patterns before they spread to the wider population.	☐
d)	All of the above	☐

H	SCHOLAR	
**28.**	**Which of the following factors is <u>not</u> included as a determinant of migrant health and inequalities?**	
a)	Disease susceptibility	☐
b)	Social stratification	☐
c)	Barriers to health services	☐
d)	Mentorship	☐
**29.**	**What approach is useful in developing evidence-based recommendations?**	
a)	GRADE (Grading of Recommendations Assessment, Development & Evaluation)	☐
b)	SOPs (Standard Operating Procedures)	☐
c)	ICRC (International Committee of the Red Cross)	☐
d)	LEARN (Listen, Explain, Acknowledge, Recommend, Negotiate)	☐
**30.**	**Which two parasites warrant routine serology testing in certain sub-populations?**	
a)	*Strongyloides* & *Ascaris lumbricoides*	☐
b)	*Schistosoma* & *Ancylostoma duodenale*	☐
c)	*Schistosoma* & *Strongyloides*	☐
d)	*Strongyloides* & *Enterobius vermicularis*	☐

<u>Comments:</u>

Thank you very much for completing this learning quiz.
