# Supplementary material for: Introducing global health into the undergraduate medical school curriculum using an e-learning program: a mixed method pilot study
Source: BMC Med Educ. 2015 Sep 2;15:142. doi: 10.1186/s12909-015-0421-3 (PMC4557599; doi:10.1186/s12909-015-0421-3)
Supplement: Additional file 3: — Focus groups questions (questions used for the qualitative component of the study). (PDF 103 kb) [file 12909_2015_421_MOESM3_ESM.pdf]

Additional file 3: Focus Group Questions

Hello/Introduction

We appreciate you taking the time to participate in the focus group discussion today. The purpose of this focus group is to obtain information on your experience with the e-learning and pdf articles and your collective thoughts on how e-learning tools can be best integrated to enhance global health curricular activities.

Focus on Features that are/were most effective

1. We would first like to know how many of you completed the e-learning modules, read the pdf articles, or the competency-based tools or were not successful in doing any of these learning activities? If you have not completed the e-learning program, do you have intentions to complete the learning activity?

Integration into the Curriculum: Supplementary e-Learning, PDF articles, and Competency Assessment Tools

2. Is there a role in your opinion for e-learning in the GH curriculum? This could include items such as supplementary online readings as well as GH competency assessment tools.

3. How should or could e-learning modules be best integrated/implemented in your curriculum? Explain your rationale.

E-Learning Tool Challenges

4. What do you think are the challenges associated with e-learning as a tool for Refugee and Global Health curriculum?

E-Learning Tool Facilitators

5. What are the factors associated with successful completion of e-learning activities?

CanMEDS Competencies

6. What do you feel is your level of knowledge with the 7 CanMEDS competencies?

Outcome

7. If you have completed the e-learning program, do you feel the e-learning modules helped you prepare your career goals? If so, how?

Open discussion to questions and/or comments

Thank you for your time!
